# Explainable artificial intelligence for predicting red blood cell transfusion in geriatric patients undergoing hip arthroplasty: Machine learning analysis using national health insurance data

**DOI:** 10.1097/MD.0000000000036909

**Published:** 2024-02-23

**Authors:** Hyunyoung Seong, Kwang-Sig Lee, Yumin Choi, Donghyun Na, Jaewoo Kim, Hyeon Ju Shin, Ki Hoon Ahn

**Affiliations:** a Department of Anesthesiology and Pain Medicine, Anam Hospital, Korea University College of Medicine, Seoul, Republic of Korea; b AI Center, Korea University College of Medicine, Seoul, Republic of Korea; c Korea University School of Mechanical Engineering, Seoul, Republic of Korea; d Department of Obstetrics & Gynecology, Anam Hospital, Korea University College of Medicine, Seoul, Republic of Korea.

**Keywords:** blood transfusion, hip arthroplasty, machine learning

## Abstract

This study uses machine learning and population data to analyze major determinants of blood transfusion among patients with hip arthroplasty. Retrospective cohort data came from Korea National Health Insurance Service claims data for 19,110 patients aged 65 years or more with hip arthroplasty in 2019. The dependent variable was blood transfusion (yes vs no) in 2019 and its 31 predictors were included. Random forest variable importance and Shapley Additive Explanations were used for identifying major predictors and the directions of their associations with blood transfusion. The random forest registered the area under the curve of 73.6%. Based on random forest variable importance, the top-10 predictors were anemia (0.25), tranexamic acid (0.17), age (0.16), socioeconomic status (0.05), spinal anesthesia (0.05), general anesthesia (0.04), sex (female) (0.04), dementia (0.03), iron (0.02), and congestive heart failure (0.02). These predictors were followed by their top-20 counterparts including cardiovascular disease, statin, chronic obstructive pulmonary disease, diabetes mellitus, chronic kidney disease, peripheral vascular disease, liver disease, solid tumor, myocardial infarction and hypertension. In terms of max Shapley Additive Explanations values, these associations were positive, e.g., anemia (0.09), tranexamic acid (0.07), age (0.09), socioeconomic status (0.05), spinal anesthesia (0.05), general anesthesia (0.04), sex (female) (0.02), dementia (0.03), iron (0.04), and congestive heart failure (0.03). For example, the inclusion of anemia, age, tranexamic acid or spinal anesthesia into the random forest will increase the probability of blood transfusion among patients with hip arthroplasty by 9%, 7%, 9% or 5%. Machine learning is an effective prediction model for blood transfusion among patients with hip arthroplasty. The high-risk group with anemia, age and comorbid conditions need to be treated with tranexamic acid, iron and/or other appropriate interventions.

## 1. Introduction

Blood transfusion is essential for 39% to 70% of geriatric patients with hip fracture surgery.^[1,2]^ Also, hip fracture surgery is the fifth most common operation requiring blood transfusion.^[3]^ However, allogeneic red blood cell transfusion is reported to cause several complications in hip fracture surgery, e.g., the growing risk of infection due to immunomodulation, delayed postoperative recovery, the increasing length of stay, the rising risk of mortality.^[2]^ Moreover, the supply of red blood cell is getting scarcer and the economic burden of blood transfusion is becoming greater. In this context, increasing effort has been made to reduce blood loss, limit blood transfusion and identify its risk factors in geriatric patients with hip fracture surgery.^[4]^ Based on existing literature, the risk factors of blood transfusion include preoperative anemia, the nonuse of tranexamic acid, age, sex (female), longer operation time and high American Society of Anesthesiologists classification.^[5–9]^

However, these previous studies suffer from limited scopes of data, predictors and methods, which result in sub-optimal clinical implications on blood transfusion among patients with hip arthroplasty.^[10,11]^ What requests due attention now is a new approach based on population data and a comprehensive set of independent variables to maximize external validity. As addressed below, machine learning (or explainable artificial intelligence) combines the results of all possible sub-group analyses, which are ignored in linear or logistic regression with an unrealistic assumption of *ceteris paribus*, i.e., “all the other variables staying constant.” In this context, this study uses machine learning, population data and 31 predictors to analyze major determinants of blood transfusion among patients with hip arthroplasty. This is expected to alleviate various complications in hip fracture surgery including the growing risk of infection due to immunomodulation, delayed postoperative recovery, the increasing length of stay and the rising risk of mortality.

## 2. Methods

### 2.1. Participants and variables

Retrospective cohort data came from Korea National Health Insurance Service claims data for 19,110 patients aged 65 years or more with hip arthroplasty arthroplasty (bipolar hemiarthroplasty, total hip arthroplasty, or revision arthroplasty) in 2019. This retrospective cohort study was approved by the Institutional Review Board of Korea University Anam Hospital on 2023.07.26 (2023AN0309). The dependent variable was blood transfusion (yes vs no) within 2 days before or after arthroplasty in 2019. The 31 independent variables covered the following information: 3 demographic/socioeconomic determinants in 2019 including age, sex (female) and low socioeconomic status measured by an insurance fee with the range of 1 (the highest socioeconomic group) to 20 (the lowest socioeconomic group); 3 anesthetic methods within 1 month from hip arthroplasty in 2019, e.g., spinal anesthesia, general anesthesia, spinal epidural anesthesia; 3 medication variables within 1 year from hip arthroplasty in 2019 including iron, antithrombotic, statin; 21 other diseases in 2019, e.g., anemia, dementia, congestive heart failure. The selection of these 31 independent variables were based on previous studies and data availability. These data of disease and medication history were screened from ICD-10 and ATC codes, respectively (Table S1, Supplemental Digital Content 1, http://links.lww.com/MD/L443).

### 2.2. Machine learning analysis

The random forest was used for the prediction of blood transfusion among analyze major determinants of blood transfusion among patients with hip arthroplasty. A random forest is a group of decision trees which make majority votes on the dependent variable (“bootstrap aggregation”). Let us take a random forest with 100 decision trees as an example. Let us assume that the original data include 20,000 participants. Then, the training and test of this random forest takes 2 steps. First, new data with 20,000 participants are created based on random sampling with the replacement, and a decision tree is created based on these new data. Here, some participants in the original data would be excluded from the new data, and these leftovers are called out-of-bag data. This process is repeated 100 times, i.e., 100 new data are created, 100 decision trees are created, and 100 out-of-bag data are created. Second, the 100 decision trees make predictions on the dependent variable of every participant in the out-of-bag data, their majority vote is taken as their final prediction on this participant, and the out-of-bag error is calculated as the proportion of wrong votes on all participants in the out-of-bag data.^[12]^ The data of 19,110 cases with full information were split into training and validation sets with an 80:20 ratio (15,288 cases vs 3822 cases). A criterion for the validation of the trained models were accuracy (a ratio of correct predictions among 3822 cases) and the area under the receiver-operating-characteristic curve (area under the plot of sensitivity vs 1 − specificity). Here, the area under the receiver-operating-characteristic curve can be interpreted as the degree of sensitivity when its threshold and specificity increase from 0 to 1.

### 2.3. Variable importance analysis

In the following context, random forest permutation importance was used for identifying major predictors of blood transfusion among patients with hip arthroplasty and Shapley Additive Explanation (SHAP) values were calculated to analyze the directions of its associations with the predictors.^[13]^ Random forest permutation importance measures the overall accuracy decrease from the permutation of data on the predictor. It is an average or sum over all trees in a random forest with the range of 0 and the number of all trees. The SHAP value of a predictor for a participant measures the difference between what machine learning predicts for the probability of transfusion with and without the predictor. For example, let us assume that the SHAP values of spinal anesthesia for transfusion have the range of (−0.05, 0.10). Here, some participants have SHAP values as low as −0.05, and other participants have SHAP values as high as 0.10. The inclusion of a predictor (spinal anesthesia) into machine learning will decrease or increase the probability of the dependent variable (transfusion) by the range of −0.05 and 0.10. In other words, there exists a positive association between spinal anesthesia and transfusion in general. Permutation importance can be considered to be a “global approach” given that it involves an “average (or sum)” overall predictions for all participants. On the other hand, the SHAP can be considered to be a “local approach” given that it involves a single prediction for a single participant.^[14]^ In practice, experts in artificial intelligence use random forest permutation importance to derive the rankings and values of all predictors for the prediction of the dependent variable. Then, they employ the SHAP plots to evaluate the directions of associations (or contributions) between the predictors and the dependent variable. Linear or logistic regression used to play this role before the SHAP approach took it over. This is because the SHAP approach has a notable strength compared to linear or logistic regression: the former considers all realistic scenarios, unlike the latter. Let us assume that there are 3 predictors of blood transfusion among patients with hip arthroplasty, i.e., anemia, age and spinal anesthesia. As defined above, the SHAP value of spinal anesthesia for transfusion for a particular participant is the difference between what machine learning predicts for the probability of transfusion with and without spinal anesthesia for the participant. Here, the SHAP value for the participant is the average of the following 4 scenarios for the participant: anemia excluded, age excluded; anemia included, age excluded; anemia excluded, age included; and anemia included, age included. In other words, the SHAP value combines the results of all possible sub-group analyses, which are ignored in linear or logistic regression with an unrealistic assumption of *ceteris paribus*, i.e., “all the other variables staying constant.” Finally, it can be noted that R-Studio 1.3.959 (R-Studio Inc.: Boston, MA) was employed for the analysis during January 1, 2023 to May 31, 2023.

## 3. Results

**Figure 1.**
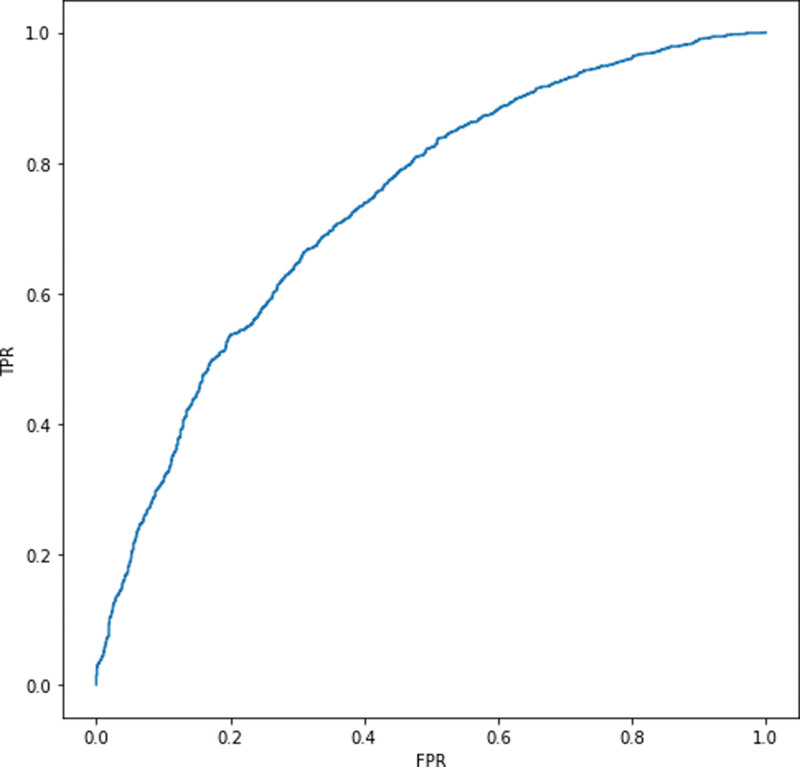
Random forest area under the curve.

**Figure 2.**
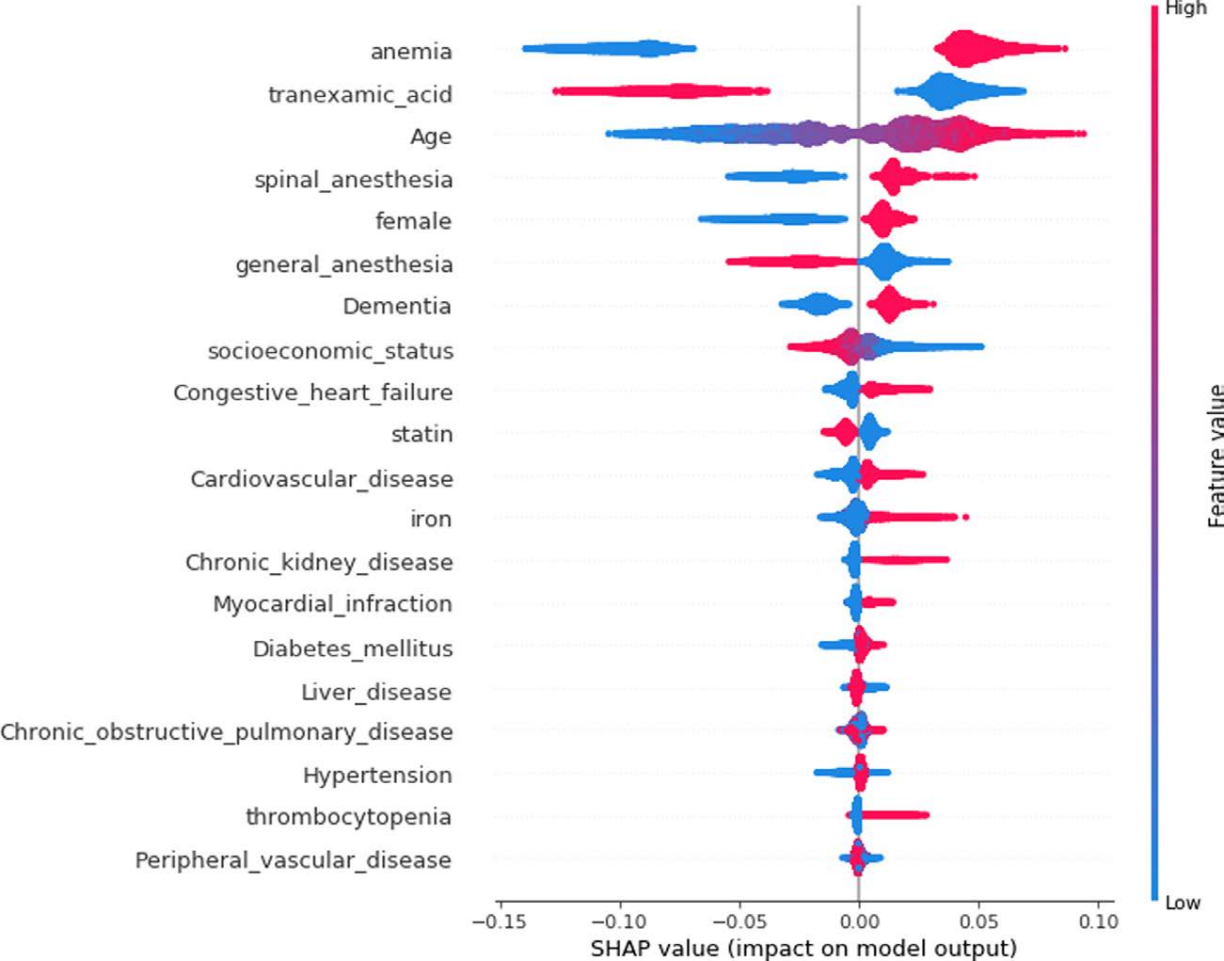
SHAP summary plot. SHAP = Shapley Additive Explanation.

Descriptive statistics for participants are given in Table 1. The proportion of blood transfusion among patients with hip arthroplasty in 2019 was 72.7% (13,894/19,110). The proportions of those with blood transfusion were higher among those with anemia and those of spinal anesthesia than among those without these risk factors, i.e., 73.4% versus 49.2% (*P* < .001) and 68.1% versus 54.8% (*P* < .001), respectively. Those with transfusion were characterized by their higher mean age and lower socioeconomic status compared to those without transfusion (80.34 years vs 77.16 years, 11.01 vs 11.74). The random forest registered the area under the curve of 73.6% (Fig. 1). Based on random forest variable importance in Table 1, the top-10 predictors were anemia (0.25), tranexamic acid (0.17), age (0.16), socioeconomic status (0.05), spinal anesthesia (0.05), general anesthesia (0.04), sex (female) (0.04), dementia (0.03), iron (0.02) and congestive heart failure (0.02). These predictors were followed by their top-20 counterparts including cardiovascular disease, statin, chronic obstructive pulmonary disease, diabetes mellitus, chronic kidney disease, peripheral vascular disease, liver disease, solid tumor, myocardial infarction and hypertension. In terms of max SHAP values in Table 2 and Figure 2, these associations were positive, e.g., anemia (0.09), tranexamic acid (0.07), age (0.09), socioeconomic status (0.05), spinal anesthesia (0.05), general anesthesia (0.04), sex (female) (0.02), dementia (0.03), iron (0.04) and congestive heart failure (0.03). For example, the inclusion of anemia, age, tranexamic acid or spinal anesthesia into the random forest will increase the probability of blood transfusion among patients with hip arthroplasty by 9%, 7%, 9% or 5%.

**Table 1:** Descriptive statistics and random forest variable importance.

Variable	All		G1		G2		*P* value	VI
	Yes	Yes %	Yes	Yes %	Yes	Yes %		
Anemia	12,816	67.06	10,251	73.78	2565	49.18	<.001	0.248
Tranexamic acid	6248	32.69	3752	27.00	2496	47.85	<.001	0.173
Age (mean)	79.47		80.34		77.16		<.001	0.159
Socioeconomic status (mean)	11.21		11.01		11.74		<.001	0.051
Spinal anesthesia	12,314	64.44	9456	68.06	2858	54.79	<.001	0.046
General anesthesia	5975	31.27	3870	27.85	2105	40.36	<.001	0.044
Sex (female)	13,852	72.49	10,463	75.31	3389	64.97	<.001	0.038
Dementia	10,459	54.73	8111	58.38	2348	45.02	<.001	0.034
Iron	4908	25.68	3729	26.84	1179	22.60	<.001	0.020
Congestive heart failure	6614	34.61	5103	36.73	1511	28.97	<.001	0.019
Cardiovascular disease	8720	45.63	6604	47.53	2116	40.57	<.001	0.018
Statin	8606	45.03	6136	44.16	2470	47.35	<.001	0.013
Chronic obstructive pulmonary disease	8667	45.35	6348	45.69	2319	44.46	.132	0.013
Diabetes mellitus	13,466	70.47	9923	71.42	3543	67.93	<.001	0.012
Chronic kidney disease	2116	11.07	1674	12.05	442	8.47	<.001	0.011
Peripheral vascular disease	12,208	63.88	8887	63.96	3321	63.67	.719	0.010
Liver disease	14,176	74.18	10,350	74.49	3826	73.35	.112	0.010
Solid tumor	4671	24.44	3414	24.57	1257	24.10	.510	0.010
Myocardial infarction	4543	23.77	3442	24.77	1101	21.11	<.001	0.010
Hypertension	16,017	83.81	11,785	84.82	4232	81.13	<.001	0.010
Peptic ulcer disease	5773	30.21	4249	30.58	1524	29.22	.070	0.009
Hypothyroidism	3315	17.35	2444	17.59	871	16.70	.153	0.008
Antithrombotic	16,850	88.17	12,263	88.26	4587	87.94	.558	0.007
Thrombocytopenia	1220	6.38	957	6.89	263	5.04	<.001	0.007
Hemiplegia	1315	6.88	985	7.09	330	6.33	.068	0.005
Connective tissue disease	829	4.34	588	4.23	241	4.62	.257	0.005
Thyrotoxicosis hyperthyroidism	1302	6.81	958	6.90	344	6.60	.483	0.004
Spinal epidural anesthesia	523	2.74	390	2.81	133	2.55	.357	0.004
Lymphoma	180	0.94	139	1.00	41	0.79	.200	0.001
Leukemia	40	0.21	24	0.17	16	0.31	.104	0.000
Acquired immune deficiency syndrome	56	0.29	31	0.22	25	0.48	.006	0.000

Values are presented as median (Q1, Q3) or frequency (%).

G1 = blood transfusion Yes (13,894 patients), G2 = blood transfusion No (5216 patients), VI = variable importance.

**Table 2:** Shapley Additive Explanation values.

Variable	Max	Min
Age (mean)	0.094	−0.105
Anemia	0.086	−0.139
Tranexamic acid	0.069	−0.127
Socioeconomic status (mean)	0.051	−0.029
Spinal anesthesia	0.048	−0.055
Iron	0.045	−0.016
General anesthesia	0.037	−0.054
Chronic kidney disease	0.037	−0.006
Dementia	0.031	−0.032
Spinal epidural anesthesia	0.030	−0.002
Congestive heart failure	0.030	−0.014
Thrombocytopenia	0.028	−0.004
Cardiovascular disease	0.027	−0.017
Female	0.023	−0.066
Myocardial infraction	0.014	−0.005
Solid tumor	0.012	−0.008
Hypertension	0.012	−0.018
Statin	0.012	−0.015
Liver disease	0.011	−0.006
Diabetes mellitus	0.010	−0.016
Chronic obstructive pulmonary disease	0.010	−0.009
Hypothyroidism	0.010	−0.009
Peptic ulcer disease	0.009	−0.006
Thyrotoxicosis hyperthyroidism	0.009	−0.011
Peripheral vascular disease	0.009	−0.007
Hemiplegia	0.009	−0.011
Antithrombotic	0.007	−0.018
Lymphoma	0.004	−0.012
Connective Tissue disease	0.002	−0.025
Aids	0.000	−0.007
Leukemia	0.000	−0.025

**Figure 3.**
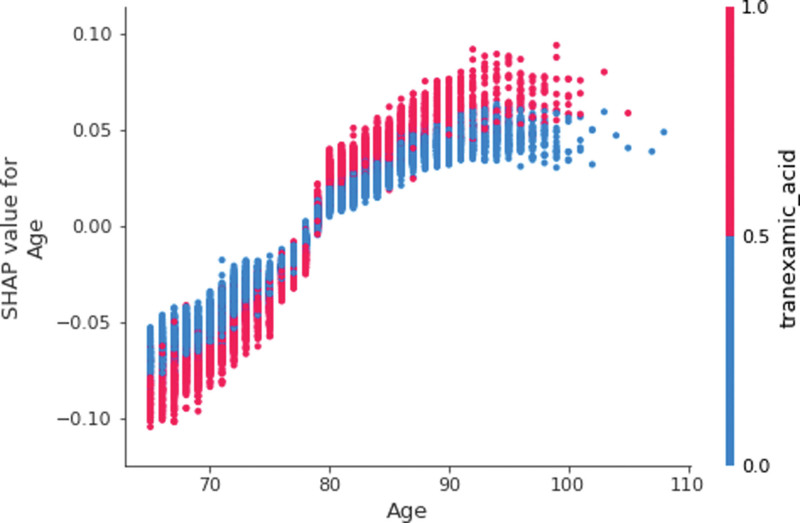
SHAP dependence plot for age. SHAP = Shapley Additive Explanation.

**Figure 4.**
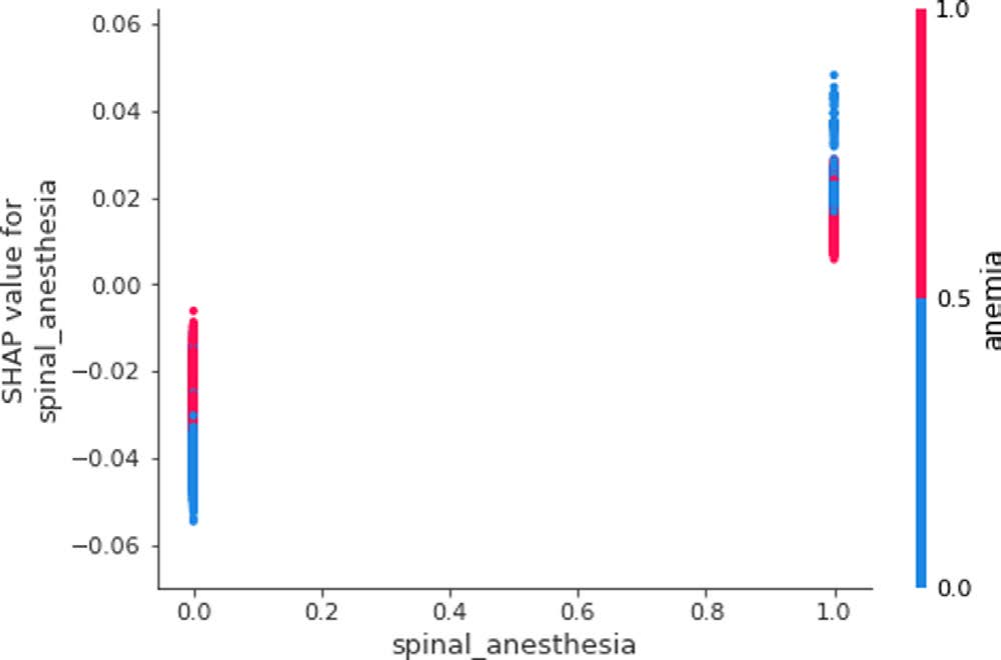
SHAP dependence plot for spinal anesthesia. SHAP = Shapley Additive Explanation.

**Figure 5.**
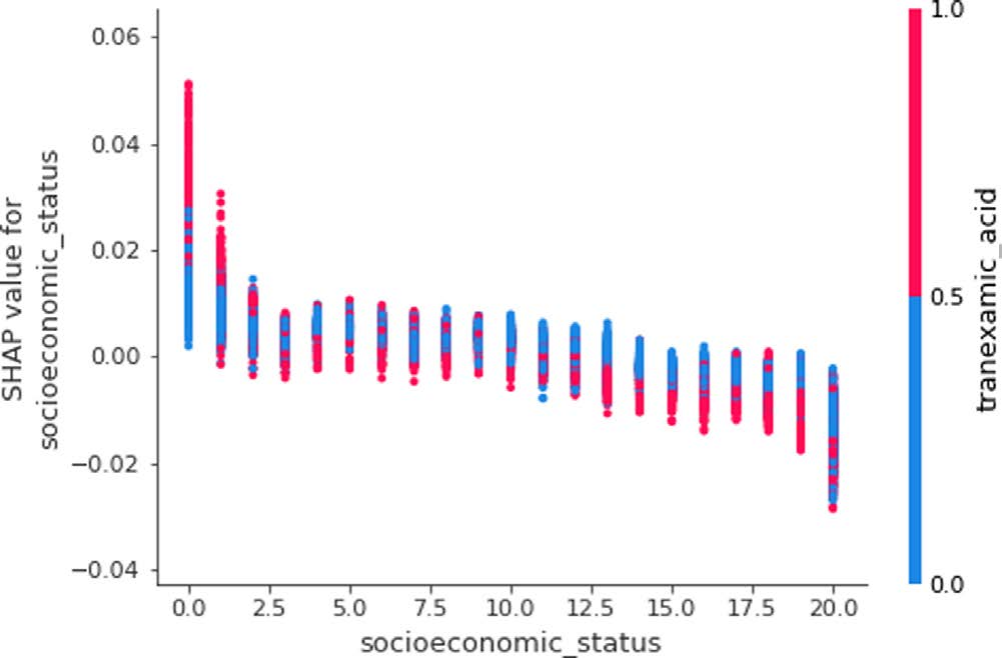
SHAP dependence plot for socioeconomic status. SHAP = Shapley Additive Explanation.

The positive association between blood transfusion and a major risk factor, age, was more apparent in Figure 3, so called a dependence plot. In this figure, the horizontal axis denotes the value of age, whereas the vertical axis denotes the SHAP value of age for blood transfusion for a particular point (a particular patient). Here, points with low values of age (i.e., <80 years) were placed in the left bottom with low SHAP values, while points with high values of age (i.e., more than 80 years) were placed in the right top with high SHAP values. That is, there exists a positive relationship between age and blood transfusion. In this figure, the blue (or red) color represents the absence (or presence) of tranexamic acid for a particular point. Tranexamic acid was chosen because it was found to have the highest contribution with age for the prediction of blood transfusion. Here, before the age of 80, the location of blue points with the absence of tranexamic acid was above the location of red points with the presence of tranexamic acid (The SHAP values of the former points were higher than those of the latter). In Figure 4, likewise, points with the absence of spinal anesthesia (i.e., the values of 0) were positioned in the left bottom with low SHAP values, whereas points with the presence of spinal anesthesia (i.e., the values of 1) were positioned in the right top with high SHAP values. In this figure, the blue (or red) color represents the absence (or presence) of anemia for a particular point, which had the highest contribution with spinal anesthesia for the prediction of blood transfusion. Here, the location of blue points with the absence of spinal anesthesia and the absence of anemia was the left bottom with low SHAP values for blood transfusion, but the location of red points with the presence of spinal anesthesia and the presence of anemia was the right top with high SHAP values for blood transfusion. The SHAP dependence plots of other predictors versus blood transfusion are presented in Figure 5 and Figures S1 to S26, Supplemental Digital Content 1, http://links.lww.com/MD/L445, Supplemental Digital Content 2, http://links.lww.com/MD/L447, Supplemental Digital Content 3, http://links.lww.com/MD/L449, Supplemental Digital Content 4, http://links.lww.com/MD/L450, Supplemental Digital Content 5, http://links.lww.com/MD/L451, Supplemental Digital Content 6, http://links.lww.com/MD/L452, Supplemental Digital Content 7, http://links.lww.com/MD/L453, Supplemental Digital Content 8, http://links.lww.com/MD/L454, Supplemental Digital Content 9, http://links.lww.com/MD/L455, Supplemental Digital Content 10, http://links.lww.com/MD/L456, Supplemental Digital Content 11, http://links.lww.com/MD/L457, Supplemental Digital Content 12, http://links.lww.com/MD/L458, Supplemental Digital Content 13, http://links.lww.com/MD/L459, Supplemental Digital Content 14, http://links.lww.com/MD/L460, Supplemental Digital Content 15, http://links.lww.com/MD/L462, Supplemental Digital Content 16, http://links.lww.com/MD/L464, Supplemental Digital Content 17, http://links.lww.com/MD/L466, Supplemental Digital Content 18, http://links.lww.com/MD/L468, Supplemental Digital Content 19, http://links.lww.com/MD/L470, Supplemental Digital Content 20, http://links.lww.com/MD/L472, Supplemental Digital Content 21, http://links.lww.com/MD/L473, Supplemental Digital Content 22, http://links.lww.com/MD/L474, Supplemental Digital Content 23, http://links.lww.com/MD/L476, Supplemental Digital Content 24, http://links.lww.com/MD/L478, Supplemental Digital Content 25, http://links.lww.com/MD/L479, Supplemental Digital Content 26, http://links.lww.com/MD/L480. Indeed, the SHAP dependence plots of socioeconomic status and spinal anesthesia only were included as Figures S27 and S28, Supplemental Digital Content 27, http://links.lww.com/MD/L481, Supplemental Digital Content 28, http://links.lww.com/MD/L483. Here, points with low values of socioeconomic status (i.e., 0) were placed in the left top with high SHAP values, while points with high values of socioeconomic status (i.e., 20) were placed in the right bottom with low SHAP values. Other predictors are not considered here. Likewise, points with the absence of spinal anesthesia (i.e., the values of 0) were positioned in the left bottom with low SHAP values, whereas points with the presence of spinal anesthesia (i.e., the values of 1) were positioned in the right top with high SHAP values. Other predictors are not considered here.

## 4. Discussion

In summary, the random forest registered a good performance measure (the area under the curve of 73.6%). Based on random forest variable importance, the top-10 predictors were anemia, tranexamic acid, age, socioeconomic status, spinal anesthesia, general anesthesia, sex (female), dementia, iron and congestive heart failure. In terms of max SHAP values, these associations were positive, e.g., the inclusion of anemia, age, tranexamic acid or spinal anesthesia into the random forest will increase the probability of blood transfusion among patients with hip arthroplasty by 9%, 7%, 9% or 5%. This study used machine learning (explainable artificial intelligence), population data and 31 predictors to analyze major determinants of blood transfusion among patients with hip arthroplasty. The following national policy can be drawn: The high-risk group with anemia, age and comorbid conditions need to be treated with tranexamic acid, iron and/or other appropriate interventions. This is expected to reduce a variety of complications in hip fracture surgery such as the growing risk of infection due to immunomodulation, delayed postoperative recovery, the increasing length of stay and the rising risk of mortality. To our best knowledge, this is one of the earliest achievements to introduce a cutting-edge approach of explainable artificial intelligence.

Specifically, 3 comments are available in the context of existing literature. Firstly, this study confirmed the great performance of the random forest as a prediction model for blood transfusion among patients with arthroplasty. One previous study employed 15,187 patients from 26 medical centers, 13 predictors and the random forest to achieve the area under the curve of 84.0% for the prediction of allogenic blood transfusion in primary lower limb total joint arthroplasty.^[10]^ On the other hand, this study utilized population data and a more comprehensive set of 31 predictors including 3 anesthetic methods within 1 month from hip arthroplasty in 2019, e.g., spinal anesthesia, general anesthesia, spinal epidural anesthesia. The results of this study are expected to deliver clinical implications with greater external validity compared to those of existing literature.

Secondly, the findings of this study agreed with those of previous studies on the positive associations of blood transfusion with age, anemia and comorbid conditions.^[8,10,15–17]^ As age increases, reportedly, the threshold of transfusion, the risk of anemia and the number of comorbid conditions are likely to increase as well.^[8,10,15]^ Based on several reviews, tranexamic acid reduces blood transfusion without promoting thromboembolic events such as deep vein thrombosis and pulmonary embolism.^[18–20]^ This result is consistent with the SHAP summary plot (Fig. 2) and the part of the SHAP dependence plot before the age of 80 (Fig. 3) in this study. Before the age of 80 in Figure 3, the location of blue points with the absence of tranexamic acid was above the location of red points with the presence of tranexamic acid (The SHAP values of the former points were higher than those of the latter). However, after the age of 80 in Figure 3, the location of blue points with the absence of tranexamic acid was below the location of red points with the presence of tranexamic acid (The SHAP values of the former points were lower than those of the latter). One possible explanation is that the effects of anemia and other predictors combined were stronger than that of tranexamic acid alone, given that the SHAP value combines the results of all possible sub-group analyses as addressed above.

Thirdly, the results of this study shed new light on a positive association between blood transfusion and spinal anesthesia. The findings of existing reviews have been mixed on the direction of the relationship^[21–25]^ and this would be because the approaches of hip arthroplasty and spinal anesthesia have shown a great variety during the study period as early as the 1960s. This study used explainable artificial intelligence and recent population data to conclude on the positive association between blood transfusion and spinal anesthesia. The 25% of complications from spinal anesthesia are hemodynamic changes such as hypotension^[26]^ and its other complications include sympathetic blockade and vasodilation.^[27]^ Indeed, Korean doctors in anesthesia give great consideration for spinal anesthesia in case patients have advanced age or multiple comorbidity.^[2]^ These factors would encourage blood transfusion.

This study had some limitations. Firstly, this study did not consider operative time, intraoperative blood loss or other surgical factors. Secondly, comorbid conditions were screened from ICD-10 codes with potential biases. Thirdly, this study used population data for Koreans and the results of this study need to be applied for other populations with caution.

## 5. Conclusion

Machine learning is an effective prediction model for blood transfusion among patients with hip arthroplasty. The high-risk group with anemia, age and comorbid conditions need to be treated with tranexamic acid, iron and/or other appropriate interventions. This is expected to alleviate various complications in hip fracture surgery including the growing risk of infection due to immunomodulation, delayed postoperative recovery, the increasing length of stay and the rising risk of mortality.

## Author contributions

**Conceptualization:** Kwang-Sig Lee, Hyeon Ju Shin, Ki Hoon Ahn.

**Data curation:** Hyunyoung Seong, Kwang-Sig Lee, Yumin Choi, Hyeon Ju Shin.

**Formal analysis:** Yumin Choi, Donghyun Na, Jaewoo Kim.

**Investigation:** Hyunyoung Seong, Hyeon Ju Shin.

**Methodology:** Kwang-Sig Lee, Yumin Choi.

**Supervision:** Hyunyoung Seong, Kwang-Sig Lee, Hyeon Ju Shin, Ki Hoon Ahn.

**Validation:** Hyunyoung Seong, Donghyun Na, Jaewoo Kim, Hyeon Ju Shin.

**Visualization:** Yumin Choi, Donghyun Na, Jaewoo Kim.

**Writing – original draft:** Hyunyoung Seong, Kwang-Sig Lee, Yumin Choi, Donghyun Na, Jaewoo Kim.

**Writing – review & editing:** Hyeon Ju Shin, Ki Hoon Ahn.

## Supplementary Material


























































