# Supplementary material for: Explainable artificial intelligence for predicting red blood cell transfusion in geriatric patients undergoing hip arthroplasty: Machine learning analysis using national health insurance data
Source: Medicine (Baltimore). 2024 Feb 23;103(8):e36909. doi: 10.1097/MD.0000000000036909 (PMC11309682; doi:10.1097/MD.0000000000036909)
Supplement: Supplementary file 1 [file medi-103-e36909-s001.docx]

**Supplemental Table 1.** Disease ICD-10 & Medication ATC Codes

| **Disease-Medication-Procedure** | **Code** |
| --- | --- |
| Bipolar Hemiarthroplasty | N0715, N2710 |
| Total Hip Arthroplasty | N0711, N2070 |
| Revision Arthroplasty | N1711, N1715, N1721, N1725, N3710, N3720, N4710, N4720 |
| Acquired Immune Deficiency Syndrome | B20 B21 B22 B23 B24 |
| Anemia | D50 D51 D52 D53 D55 D56 D57 D58 D59 D60 D61 D62 D63 D64 |
| Cardiovascular Disease | I60 I61 I62 I63 I64 I65 I66 I67 I68 I69 |
| Chronic Kidney Disease | N18 |
| Chronic Obstructive Pulmonary Disease | J41 J42 J43 J44 |
| Congestive Heart Failure | I50 |
| Connective Tissue Disease | M30 M31 M32 M33 M34 M35 M36 |
| Dementia | F00 F01 F02 F03 F04 F05 F06 F07 F08 F09 |
| Diabetes Mellitus | E10 E11 E12 E13 E14 |
| Hemiplegia | G80 G81 G82 G83 |
| Hypertension | I10 I11 I12 I13 I15 O10 |
| Hypothyroidism | E02 E03 |
| Leukemia | C91 C92 C93 C94 C95 |
| Liver Disease | K70 K71 K72 K73 K74 K75 K76 K77 |
| Lymphoma | C81 C82 C83 C84 C85 C86 C87 C88 C89 C90 |
| Myocardial Infarction | I20 I21 I22 I23 I24 I25 |
| Peptic Ulcer Disease | K27 |
| Peripheral Vascular Disease | K25 K26 K27 K28 |
| Solid Tumor | C0 C1 C2 C3 C4 C5 C6 C7 C80 |
| Thrombocytopenia | D65 D693 D695 D696 |
| Thyrotoxicosis Hyperthyroidism | E05 |
| Tranexamic Acid | 242501ACH, 242531BIJ, 242530BIJ, 242532BIJ, 242502BIJ, 242503BIJ, 242504BIJ |
| Antithrombotic | 110702ATB, 652301ATB, 652302ATB, 652303ATB, 136901ATB, 136902ATB, 492501ATB, 495201ATB, 498801ATB, 498900ATB, 501501ATB, 517900ACE, 517900ATE, 667500ACE, 597301ATB, 597302ATB, 615901ATB, 615902ATB, 111001ACE, 111001ATE, 244101ACH, 24102ACH, 489700ACR, 249103ATB, 249105ATB, 511401ATB, 511402ATB, 511403ATB, 511404ACH, 511404ATB, 613701ACH, 613702ACH, 617001ATB, 617002ATB, 643601ATB, 643602ATB, 643603ATB, 116201ATB, 116202ATB, 133201ACR, 133201ATB, 133201ATR, 133202ATB, 133203ACR, 133203ATR, 174701ATB, 226101ATB, 226103ATR, 239201ATB, 239202ATB, 506100ATB, 687200ATR, 438901ATB, 512430BIJ, 512431BIJ, 512432BIJ, 100430BIJ, 100431BIJ, 223501BIJ, 223502BIJ, 240230BIJ, 359532BIJ, 359632BIJ, 359633BIJ, 450302BIJ, 140232BIJ, 140230BIJ, 140234BIJ, 140231BIJ, 140233BIJ, 152130BIJ, 152131BIJ, 152132BIJ, 152133BIJ, 152134BIJ, 168636BIJ, 168630BIJ, 168632BIJ, 168637BIJ, 168638BIJ, 168631BIJ, 198430BIJ, 198432BIJ, 450130BIJ, 465830BIJ, 465831BIJ, 650501BIJ, 164301BIJ, 461501BIJ, 461502BIJ, 109301BIJ, 635801BIJ, 471430CSI, 246401BIJ, 246405BIJ, 246406BIJ, 110701ATB, 233303ACS, 111002ATE, 111003ATE, 110801ATB, 110802ATB, 110902BIJ, 111001ATB, 111003ACE, 244101ACE, 359531BIJ, 198431BIJ, 447530BIJ, 359501BIJ, 152135BIJ, 447531BIJ, 447532BIJ, 256800ATB, 359530BIJ, 359630BIJ, 359631BIJ, 198433BIJ, 512401BIJ, 512402BIJ, 512403BIJ, 100401BIJ, 100402BIJ, 240201BIJ, 246407BIJ, 359502BIJ, 359503BIJ, 359601BIJ, 359602BIJ, 359603BIJ, 140201BIJ, 140202BIJ, 140203BIJ, 152101BIJ, 152102BIJ, 152103BIJ, 152104BIJ, 152105BIJ, 152106BIJ, 168601BIJ, 168602BIJ, 198401BIJ, 198402BIJ, 198403BIJ, 198407BIJ, 447501BIJ, 447502BIJ, 447503BIJ, 450101BIJ, 465801BIJ, 465802BIJ, 471401CSI, 246404BIJ, 450301BIJ, 147201ATB, 446301BIJ, 446302BIJ, 446303BIJ, 446304BIJ, 424401BIJ, 147203ATB, 111001ACH, 168603BIJ, 168605BIJ, 168606BIJ, 168607BIJ |
| Iron | 158631ALQ, 158634ALQ, 158632ALQ, 215001ACH, 228902ACH, 303403ATR, 379330ALQ, 379302ACH, 533700ALQ, 529300ALQ, 529400ALQ, 529500ASS, 529600ASS, 359730BIJ, 359731BIJ, 228931ALQ, 303300ATB, 417230ASY, 215002ASY, 177530BIJ, 158630ALQ, 158633ALQ, 158635ALQ, 158636ALQ, 158637ALQ, 177602ATB, 177630ALQ, 215003ATB, 367602ATB, 529200ALQ, 228930ALQ, 463501AGN, 463502AGN, 463501ACH, 158530BIJ, 215030ASY, 215031ASY, 158601ALQ, 158602ALQ, 158603ALQ, 177601ALQ, 215005ASY, 228903ALQ, 228904ALQ, 303800ALQ, 379301ALQ, 417201ASY, 304700ALQ, 365700ALQ, 502800ALQ, 515100ASS, 158501BIJ, 177501BIJ, 359701BIJ, 359702BIJ, 229001ATB, 367601ATB |
| Statin | 111501ATB, 111502ATB, 111503ATB, 111504ATB, 162401ACH, 162402ACH, 162403ATR, 185801ATB, 216601ATB, 216602ATB, 216603ATB, 216604ATB, 227801ATB, 227801ATR, 227802ATB, 227806ATB, 454001ATB, 454001ATD, 454002ATB, 454002ATD, 454003ATB, 454003ATD, 454005ATB, 470901ATB, 470902ATB, 470903ATB, 471000ATB, 471100ATB, 507800ATB, 502201ATB, 502202ATB, 502203ATB, 502204ATB, 519300ACH, 633800ATB, 633900ATB, 634600ATB, 634800ATB, 640700ATB, 640800ATB, 640900ATB, 701100ATB, 663400ACS, 679300ACH, 694000ACS, 699400ATB, 699500ATB, 472300ATB, 472400ATB, 472500ATB, 518900ATB, 524000ATB, 524100ATB, 527000ATB, 527100ATB, 525000ATB, 525100ATB, 525200ATB, 525300ATB, 629700ATB, 629800ATB, 526300ATB, 526400ATB, 526500ATB, 526900ATB, 644100ATB, 644200ATB, 653200ATB, 614500ATB, 629900ATB, 630000ATB, 630100ATB, 630200ATB, 631600ATB, 631700ATB, 634900ATB, 635000ATB, 635100ATB, 635200ATB, 654700ATB, 654800ATB, 654900ATB, 655000ATB, 661800ATB, 661900ATB, 662000ATB, 662100ATB, 673700ATB, 663900ATB, 664000ATB, 664100ATB, 664200ATB, 664300ATB, 664400ATB, 671200ATB, 671300ATB, 671400ATB, 671500ATB, 671600ATB, 671700ATB, 677000ATB, 677100ATB, 673900ATB, 674000ATB, 674100ATB, 678600ATB, 677300ATB, 677400ATB, 677500ATB, 686800ATB, 686900ATB, 679500ATB, 679600ATB, 679700ATB, 680300ATB, 691400ATB, 691500ATB, 683000ATB, 683100ATB, 683200ATB, 691200ATB, 693000ATB, 708900ATB, 684300ATB, 684400ATB, 684500ATB, 684600ATB, 684700ATB, 688100ATB, 688200ATB, 688300ATB, 688400ATB, 688500ATB, 690400ATB, 690500ATB, 690600ATB, 690700ATB, 692000ATB, 692100ATB, 692200ATB, 692300ATB, 692400ATB, 692500ATB, 701800ATB, 701900ATB, 702100ATB, 702200ATB, 702000ATB, 706300ATB, 706400ATB, 706500ATB, 706600ATB, 706700ATB, 664600ATB, 664700ATB, 664800ATB, 671800ATR, 673800ATR, 671900ATR, 672000ATR, 672100ATR, 672500ATR, 672600ATR, 672700ATR, 672800ATR, 672900ATR, 683300ATR, 683400ATR, 631400ATB, 631500ATB, 677600ATB, 673000ATR, 654600ATB, 227803ATB, 227805ATB |
| General Anesthesia | L1211 |
| Spinal Anesthesia | L1213 |
| Spinal Epidural Anesthesia | L1216 |
