# Supplementary figures and images for: Explainable artificial intelligence for predicting red blood cell transfusion in geriatric patients undergoing hip arthroplasty: Machine learning analysis using national health insurance data
Source: Medicine (Baltimore). 2024 Feb 23;103(8):e36909. doi: 10.1097/MD.0000000000036909 (PMC11309682; doi:10.1097/MD.0000000000036909)

**Supplemental Figure 1.** SHAP Dependence Plot for General Anesthesia


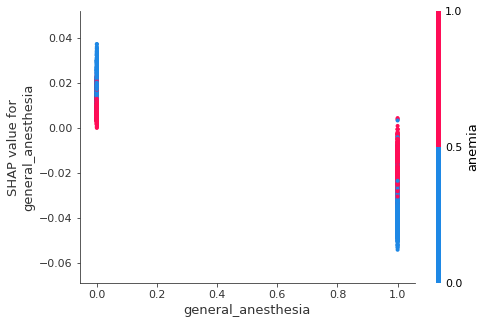

Supplement: Supplementary file 2 [file medi-103-e36909-s002.docx]

**Supplemental Figure 2.** SHAP Dependence Plot for Tranexamic acid


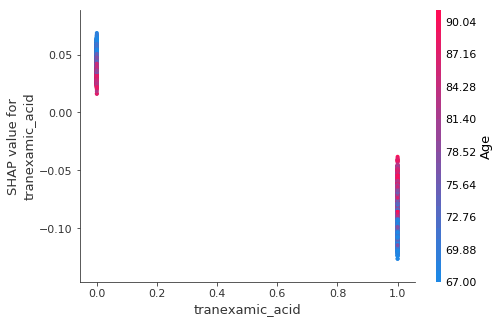

Supplement: Supplementary file 3 [file medi-103-e36909-s003.docx]

**Supplemental Figure 3.** SHAP Dependence Plot for Sex (female)


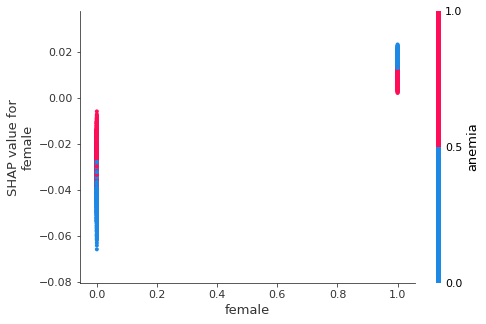

Supplement: Supplementary file 4 [file medi-103-e36909-s004.docx]

**Supplemental Figure 4.** SHAP Dependence Plot for Dementia


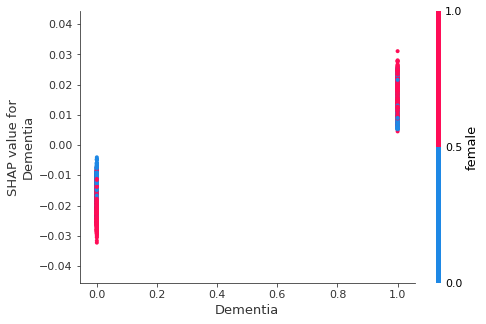

Supplement: Supplementary file 5 [file medi-103-e36909-s005.docx]

**Supplemental Figure 5.** SHAP Dependence Plot for Iron


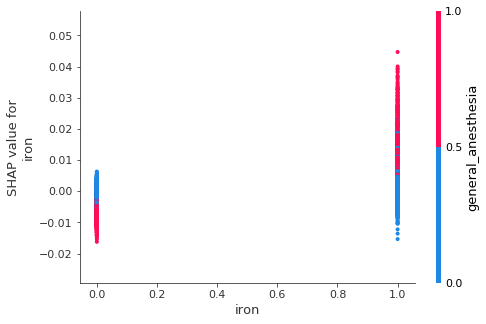

Supplement: Supplementary file 6 [file medi-103-e36909-s006.docx]

**Supplemental Figure 6.** SHAP Dependence Plot for Congestive heart failure


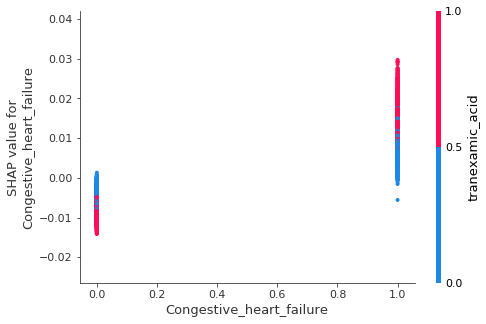

Supplement: Supplementary file 7 [file medi-103-e36909-s007.docx]

**Supplemental Figure 7.** SHAP Dependence Plot for Cardiovascular disease


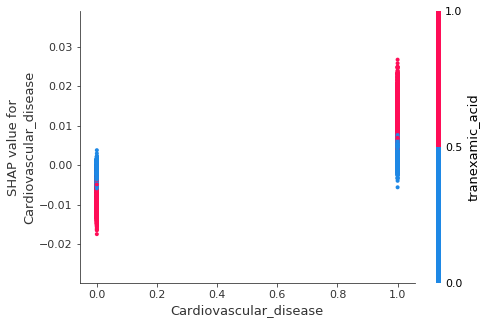

Supplement: Supplementary file 8 [file medi-103-e36909-s008.docx]

**Supplemental Figure 8.** SHAP Dependence Plot for Statin


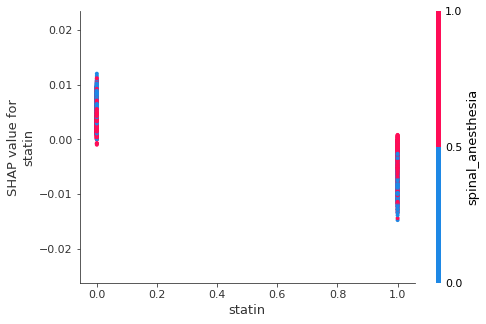

Supplement: Supplementary file 9 [file medi-103-e36909-s009.docx]

**Supplemental Figure 9.** SHAP Dependence Plot for Chronic obstructive pulmonary disease


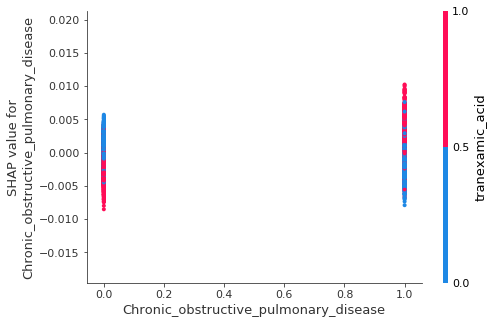

Supplement: Supplementary file 10 [file medi-103-e36909-s010.docx]

**Supplemental Figure 10.** SHAP Dependence Plot for Diabetes mellitus


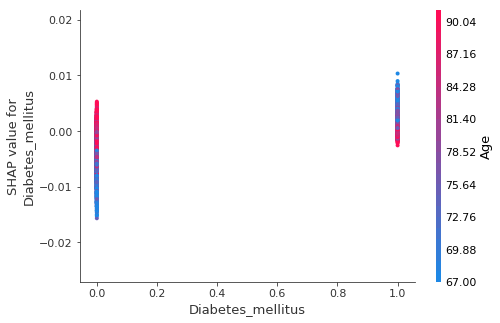

Supplement: Supplementary file 11 [file medi-103-e36909-s011.docx]

**Supplemental Figure 11.** SHAP Dependence Plot for Chronic kidney disease


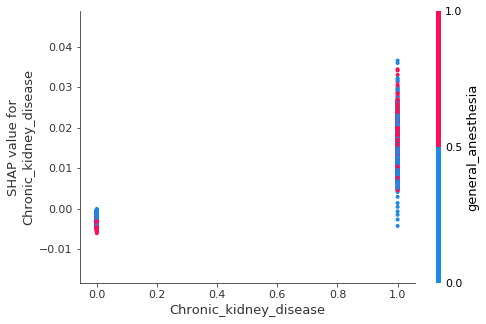

Supplement: Supplementary file 12 [file medi-103-e36909-s012.docx]

**Supplemental Figure 12.** SHAP Dependence Plot for Peripheral vascular disease


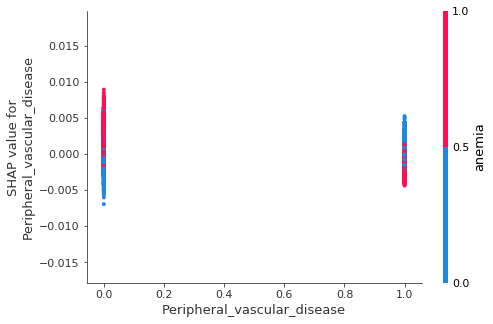

Supplement: Supplementary file 13 [file medi-103-e36909-s013.docx]

**Supplemental Figure 13.** SHAP Dependence Plot for Liver disease


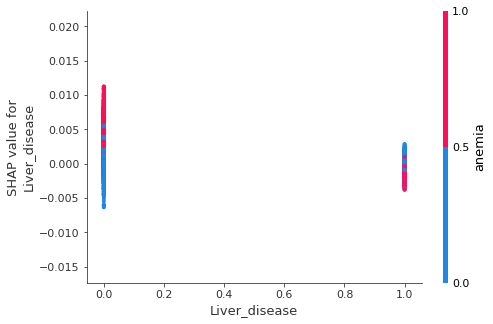

Supplement: Supplementary file 14 [file medi-103-e36909-s014.docx]

**Supplemental Figure 14.** SHAP Dependence Plot for Solid tumor


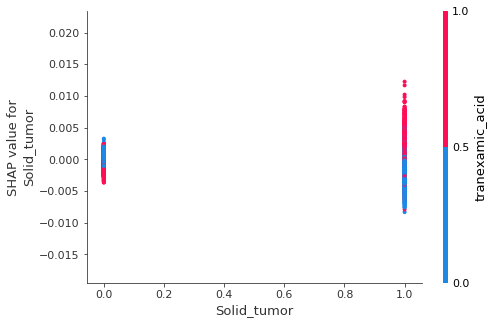

Supplement: Supplementary file 15 [file medi-103-e36909-s015.docx]

**Supplemental Figure 15.** SHAP Dependence Plot for Myocardial infarction


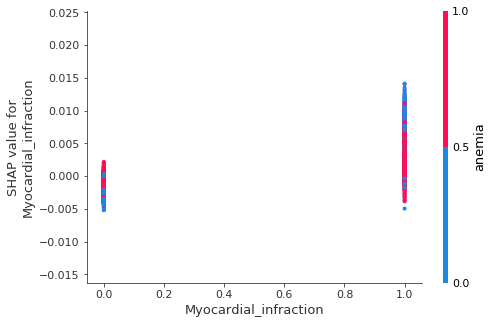

Supplement: Supplementary file 16 [file medi-103-e36909-s016.docx]

**Supplemental Figure 16.** SHAP Dependence Plot for Hypertension


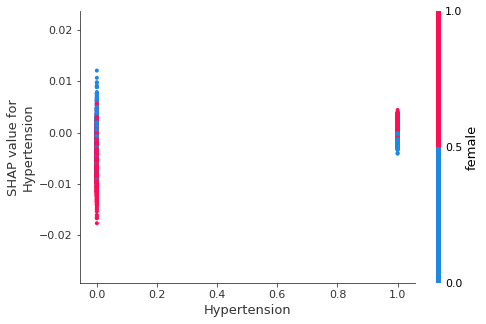

Supplement: Supplementary file 17 [file medi-103-e36909-s017.docx]

**Supplemental Figure 17.** SHAP Dependence Plot for Peptic ulcer disease


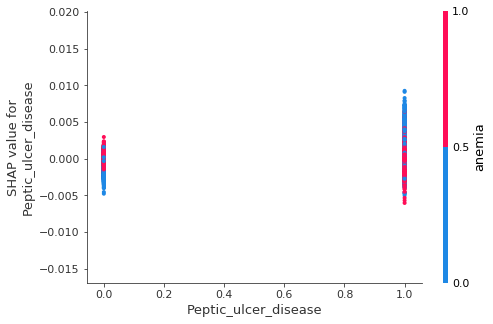

Supplement: Supplementary file 18 [file medi-103-e36909-s018.docx]

**Supplemental Figure 18.** SHAP Dependence Plot for Hypothyroidism


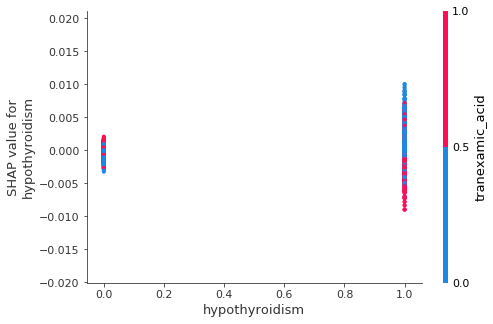

Supplement: Supplementary file 19 [file medi-103-e36909-s019.docx]

**Supplemental Figure 19.** SHAP Dependence Plot for Antithrombotic agents


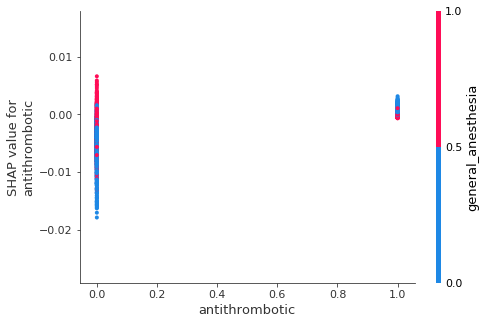

Supplement: Supplementary file 20 [file medi-103-e36909-s020.docx]

**Supplemental Figure 20.** SHAP Dependence Plot for Thrombocytopenia


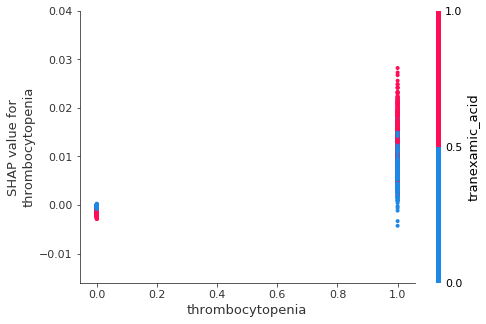

Supplement: Supplementary file 21 [file medi-103-e36909-s021.docx]

**Supplemental Figure 21.** SHAP Dependence Plot for Hemiplegia


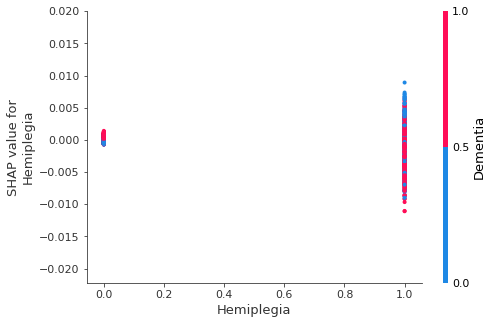

Supplement: Supplementary file 22 [file medi-103-e36909-s022.docx]

**Supplemental Figure 22.** SHAP Dependence Plot for Connective tissue disease


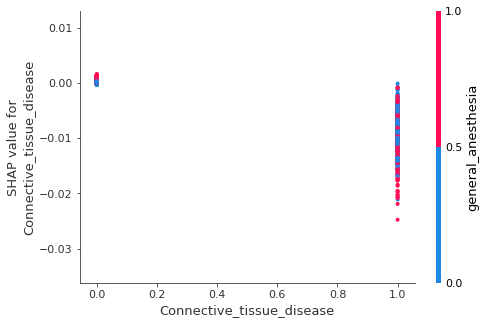

Supplement: Supplementary file 23 [file medi-103-e36909-s023.docx]

**Supplemental Figure 23.** SHAP Dependence Plot for Hyperthyroidism


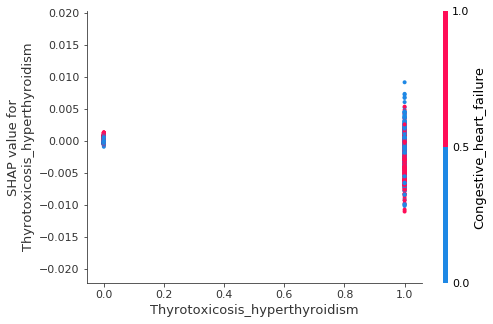

Supplement: Supplementary file 24 [file medi-103-e36909-s024.docx]

**Supplemental Figure 24.** SHAP Dependence Plot for Lymphoma


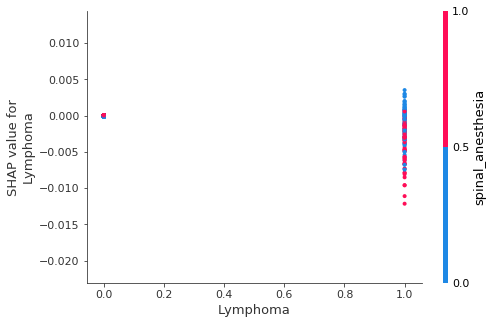

Supplement: Supplementary file 25 [file medi-103-e36909-s025.docx]

**Supplemental Figure 25.** SHAP Dependence Plot for Leukemia


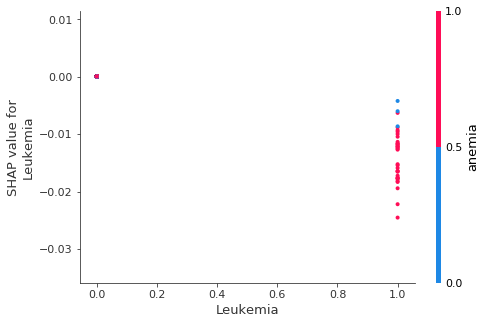

Supplement: Supplementary file 26 [file medi-103-e36909-s026.docx]

**Supplemental Figure 26.** SHAP Dependence Plot for AIDS


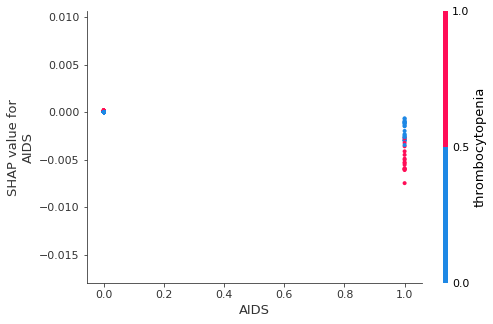

Supplement: Supplementary file 27 [file medi-103-e36909-s027.docx]

**Supplemental Figure 28.** SHAP Dependence Plot for spinal anesthesia


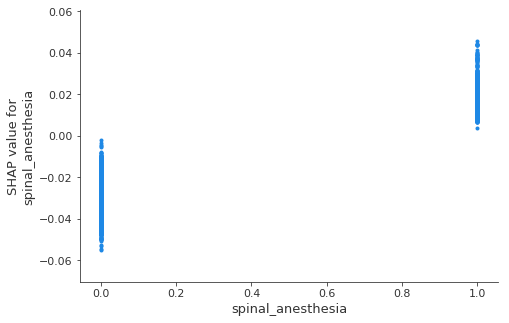

Supplement: Supplementary file 28 [file medi-103-e36909-s028.docx]

**Supplemental Figure 27.** SHAP Dependence Plot for socioecomonic status


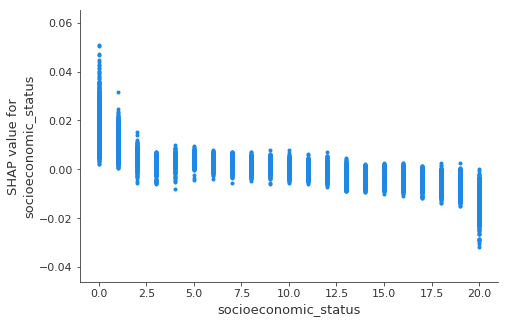

Supplement: Supplementary file 29 [file medi-103-e36909-s029.docx]
